# The Impact of Incidental Fear on Empathy Towards In-Group and Out-Group Pain

**DOI:** 10.3390/bs15091186

**Published:** 2025-08-30

**Authors:** Binghai Sun, Weihao Chi, Weihao Ye, Tinghui Dai, Yaoyao Wang

**Affiliations:** School of Psychology, Zhejiang Normal University, Jinhua 321004, China

**Keywords:** empathy, fear, intergroup relations, pain, incidental emotion

## Abstract

Fear modulates intergroup dynamics by amplifying biases, yet prior work predominantly examines integrated emotions (e.g., fear tied to intergroup conflict), neglecting incidental fear—transient states unrelated to group contexts. Furthermore, the reliance on Western samples limits insights into cultural variability, particularly in collectivist societies where group boundaries differ. Here, we conducted two experimental studies that involved Chinese participants and examined the effect of incidental fear on empathy for in-group and out-group members’ pain (operationalized as self-focused empathy, reflecting personal distress when witnessing pain, and other-focused empathy, reflecting compassionate concern for the sufferer). In Experiment 1 (*N* = 54), using a pain observation paradigm, incidental fear was elicited by randomly showing frightening images, while the differentiation between the in-group and out-group was based on natural ethnic differences (same races and other races). Experiment 2 (*N* = 52) replicated this using artificial social categorization (university affiliation). Fear reduced other-focused empathy for racial out-groups and socially defined out-groups. Self-focused empathy remained unaffected, suggesting fear selectively disrupts mentalizing-dependent processes. The Inclusion of Other in Self (IOS) scale revealed heightened psychological distance toward out-groups under fear, mediating empathy reduction. Incidental fear universally diminishes empathy for out-group pain across natural and artificial group boundaries, extending social identity theory to transient affective states. These findings highlight fear’s role in intergroup bias and underscore cultural generalizability beyond WEIRD populations.

## 1. Introduction

Pain empathy—the perception, evaluation, and emotional response to others’ suffering—varies contextually and individually (Smith et al., 2021; Stietz et al., 2019). A robust phenomenon, intergroup empathy bias, describes attenuated empathy when observers perceive sufferers as out-group members (e.g., differing ethnicity, religion) (Bruneau et al., 2017; Han, 2018; Hein et al., 2010). While negative emotions like fear exacerbate out-group stereotypes (Mekawi et al., 2016; Rösler & Amodio, 2022), research focuses on integrated emotions (affective states tied to group contexts;, e.g., fear arising from intergroup conflict), overlooking incidental emotions—transient states arising independently of intergroup dynamics (e.g., fear triggered by spiders or sharks) (Kruglanski, 2010; Vohs et al., 2007). Crucially, whether incidental fear similarly biases empathy in non-Western, collectivist contexts remains untested, despite evidence that cultural norms (e.g., in-group cohesion in East Asia) may modulate threat responses and empathy (Gelfand et al., 2011). This gap limits the cross-cultural validity of prevailing theories, which predominantly derive from Euro-American samples. By examining Chinese participants, this study tests the universality of fear’s empathy-reducing effects while addressing the underrepresentation of Same race populations in intergroup emotion research.

As mentioned above, pain empathy exhibits a pronounced intergroup bias (Sessa et al., 2014; Sheng & Han, 2012). Neuroimaging studies confirm heightened empathic neural responses (e.g., anterior insula activation) for racial in-groups versus out-groups (Fan & Han, 2008; Azevedo et al., 2013), while minimal group paradigms reveal bias even in arbitrary categories (e.g., sports teams) (Hein et al., 2010; Huang & Han, 2014). Social identity theory posits that such categorization spontaneously triggers in-group favoritism and out-group derogation (Tajfel & Turner, 1979), with empathy bias reflecting defensive prioritization of “us” over “them.” However, this framework neglects emotional moderators—particularly fear—that may amplify or reconfigure these biases.

Extensive research has documented that emotional states, across valence and arousal levels, exert profound influences on empathic responses. Positive emotional states, such as happiness and compassion, generally enhance empathic concern and perspective-taking by broadening cognitive scope and fostering perceived similarity with others (Fredrickson, 2004). For instance, induced positive affect has been shown to reduce intergroup empathy gaps by promoting more inclusive social categorizations (Tugade & Fredrickson, 2004). In contrast, negative emotions exhibit more nuanced effects: while sadness can amplify empathic distress by increasing sensitivity to others’ suffering, anger often narrows attention toward threat-related cues, impairing empathy for out-groups. Disgust, particularly intergroup disgust, uniquely diminishes empathy by activating dehumanizing perceptions of target groups.

Emerging evidence implicates fear as a catalyst for intergroup bias. Negative emotions, especially fear, heighten threat vigilance and psychological distancing from out-groups (Bodenhausen et al., 2001; Mackie et al., 2000; Richins et al., 2021), potentially via amygdala-driven defensive mechanisms (Hodson et al., 2013). Fear’s survival-driven nature primes associations between out-groups and danger (Navarrete et al., 2009), even absent objective threats, thereby reducing empathy (Navarrete et al., 2012). This aligns with dual-process models: fear disrupts other-focused empathy (reliant on mentalizing networks like mPFC) while sparing self-focused empathy (rooted in sensorimotor simulation) (Azevedo et al., 2013). Yet, whether such effects generalize to incidental fear—divorced from intergroup contexts—remains unexplored, a gap this study bridges.

Despite the established link between fear and group bias, existing research has predominantly focused on European and American participant samples. This gap is critical given evidence of overreliance on WEIRD (Western, Educated, Industrialized, Rich, Democratic) populations (Cheon et al., 2020; Henrich et al., 2010), which limits generalizability to non-Western settings where cultural norms shape empathy, threat responses, and intergroup boundaries (Cheon et al., 2011). For example, collectivist societies such as China emphasize in-group cohesion, potentially moderating fear’s effects on empathy biases compared to individualist contexts. By investigating Chinese participants, this study addresses the WEIRD-centric bias in the literature and tests whether fear-induced reductions in out-group empathy hold across cultural frameworks, thereby advancing universal theories of intergroup dynamics while accounting for cultural specificity.

Additionally, recent research highlights the role of emotional states, particularly negative ones, such as fear, which may exacerbate negative stereotypes towards outgroups. While research has focused on integrated emotions—emotional states directly tied to intergroup contexts or decision-making processes (e.g., fear arising from intergroup conflict or anger linked to resource competition) (Bodenhausen et al., 2001; Mackie et al., 2000)—incidental emotions, arising independently of decision-making, significantly impact heuristics and may introduce bias. For instance, integrated emotions are often studied in paradigms where emotions are elicited by group-relevant stimuli (e.g., fear of out-group members in Navarrete et al., 2009), whereas incidental emotions involve affective states induced by unrelated stimuli (e.g., fear triggered by spiders or sharks; Lerner et al., 2013) that carry over to influence unrelated judgments. This distinction is critical because incidental emotions, unlike integrated ones, operate through heuristic processes rather than deliberative appraisals (Kruglanski, 2010; Richins et al., 2021). As such, this research delves into how incidental fear influences various racial and social groups in order to build upon existing studies.

### The Current Project

To summarize, this research investigates whether incidental fear—a contextually irrelevant emotional state distinct from integrated or chronic emotions—shapes empathy bias in Chinese participants, addressing a critical gap in cultural generalizability given the predominance of Western samples in prior intergroup empathy research. To address these gaps, we posed two core research questions: (1) Does incidental fear reduce other-focused empathy for out-group pain among Chinese participants, a collectivist sample underrepresented in prior research? (2) Do these effects generalize across both natural (racial) and artificial (socially constructed) group boundaries? And we designed two experiments that collectively highlight three novel contributions to the literature.

Experiment 1 leverages Chinese participants to test the effect of incidental fear—induced via aversive images unrelated to group identity (e.g., spiders, sharks)—on empathy for racially defined Asian (same races) and African (other races) groups. This design intentionally diverges from prior work, which has primarily focused on integrated emotions (e.g., fear explicitly linked to intergroup interactions) by isolating the impact of incidental fear—a theoretically understudied construct—on pain empathy. We hypothesized that such fear, by heightening threat vigilance, would diminish other-focused empathy for out-group pain, a process indexed by the Inclusion of Other in Self (IOS) scale (Aron et al., 1992)—a validated measure of psychological distance via self-other overlap.

Experiment 2 extends this inquiry to artificial group boundaries using Tajfel and Turner’s minimal group paradigm (Tajfel & Turner, 1979). It tests whether incidental fear amplifies empathy bias in socially constructed groups (university affiliation), diverging from Richins et al. (Richins et al., 2021) by anchoring group membership in real-world institutional identities (e.g., university logos) rather than arbitrary teams. This bridges laboratory paradigms with naturalistic intergroup dynamics while examining fear’s generalizability across categorization types.

## 2. Experiment 1

### 2.1. Methods

#### 2.1.1. Participants

G*Power 3.1 was used with an ANOVA (repeated measures, within-between interaction) setting. The input parameters were as follows: f = 0.25, α = 0.05 and power = 0.80, yielding a minimum *N* = 24. To account for potential attrition and design complexity, we recruited 54 participants (24 females, aged 20.00 ± 0.21 years) in Experiment 1. Participants were undergraduate students from Zhejiang Normal University. Participants were randomly assigned to the fear group (13 females, 13 males, aged 20.59 ± 0.31 years) and the control group (11 females, 14 males, aged 20.23 ± 0.27 years). Participants were right-handed individuals with normal or corrected vision, who had no history of chronic or mental illness. All participants provided written, informed consent before beginning experimentation. Recruitment used campus bulletins, and participants received course credit.

#### 2.1.2. Stimulus

**Figure 1:**
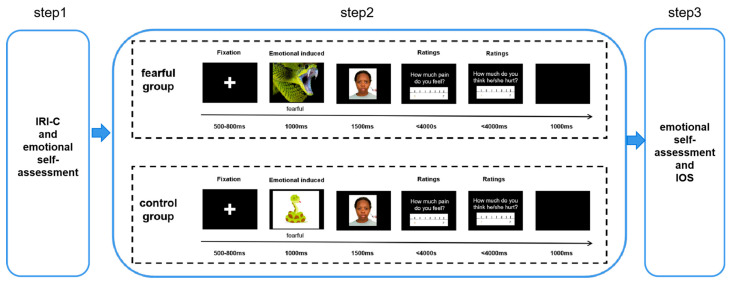
Experimental procedure. Each trial started with a fixation cross presented for 800 ms, followed by a fearful (fearful group) or non-fearful (control group) animal presented for 1000 ms. Then, a picture (painful or nonpainful) presented for 1500 ms. Participants were instructed to rate the self-focused empathy intensity (4000 ms) and other-focused empathy (4000 ms) of the picture on the 7-point numerical rating scale. The intertrial interval (ITI) was 1000 ms.

The experimental materials consisted of 40 same race and 20 other race faces with neutral expressions sourced from the Chicago Face Database (Ma et al., 2015). Image standardization was performed using Adobe Photoshop CS6. In the case of painful images, a needle was placed on the left cheek of the original face image, while non-painful images featured a cotton swab on the left cheek, as depicted in Figure 1. Fear-inducing images, such as sharks and spiders, were chosen from the International Affective Picture System (IPAS) (Lerner et al., 2013), while the control group selected equivalent cartoon animal pictures from Pixabay (https://pixabay.com/), a public domain online repository of free stock images.

Thirty-two students (16 women, aged 22.00 ± 1.98 years) who were not part of the main study participants utilized a 9-point Likert scale to evaluate the pain level, attractiveness, emotional valence, and emotional arousal of images. This independent evaluation ensured the stimuli were appropriately selected for the main experiment. In conclusion, a total of 72 images were chosen, comprising 36 depicting pain and 36 depicting non-pain (18 same race and 18 other race). These images were carefully selected to ensure uniform resolution, brightness, and contrast, each measuring 260 × 300 pixels, with an equal distribution of male and female subjects. Furthermore, participants rated both the horror and non-horror images on a scale of 1 to 9 for their perceived level of fear and emotional arousal. Subsequently, 16 images from each category were chosen for the study. Pain images were rated significantly higher in both pain intensity (pain images: 6.08 ± 1.69; non-pain images: 2.75 ± 1.48, *p* < 0.001) and arousal (pain images: 5.07 ± 1.03; non-pain images: 3.72 ± 0.89, *p* < 0.001) compared to non-pain images. There was no significant difference in arousal and pain levels observed across images depicting individuals of various races. The level of fear and emotional arousal was significantly greater in fearful images compared to non-fearful images. A total of 64 trials, 50% depicting painful events (32), and 25% per race type (16) were presented in 2 blocks.

#### 2.1.3. Measure

In order to evaluate and manage the emotional state of participants, they completed the Positive and Negative Affect Schedule (PANAS) (Lerner et al., 2013), a validated emotional self-assessment scale, prior to and after the experiment. The scale consists of seven emotional items: sadness, anger, happiness, fear, surprise, disgust, and calmness, using a seven-point scale, ranging from 1 (not at all) to 7 (very much). Emotion terms were randomized to mitigate priming effects, and participants were unaware of the study’s focus on fear until debriefing.

Participants’ empathy was measured using a Chinese translation of the Interpersonal Reactivity Index (IRI) (Davis, 1983), a self-reported questionnaire. It is composed of four subscales: empathic concern, personal distress, perspective taking and fantasy. Each subscale includes 7 items, assessed on a 5-point Likert-scale ranging from 0 (Does not describe me well) to 4 (Describes me very well). Reliability for IRI subscales was acceptable (Cronbach’s α = 0.82 for the total scale).

Psychological distance was measured using the validated Inclusion of Other in Self (IOS) scale (Aron et al., 1992), a pictorial instrument assessing perceived self-target overlap. The Inclusion of Other in Self (IOS) scale was included to measure psychological distance—a theoretical mechanism through which incidental fear might bias empathy. Comprising seven circle pairs with increasing geometric overlap between a self-representing small circle and a target-representing large circle, the scale uses a 1–7 scoring system. Scores range from 1 (completely separate circles, least overlap) to 7 (fully merged circles, maximum overlap), with lower values indicating greater psychological distance and higher values reflecting stronger self-other integration, consistent with the original IOS methodology. Psychological distance was measured using the Inclusion of Other in Self (IOS) scale (Aron et al., 1992), which demonstrated adequate reliability here (Cronbach’s α = 0.76).

#### 2.1.4. Procedure

The experiment took place in a quiet, soundproof laboratory set at an appropriate temperature. The subjects were positioned 80 cm in front of a 24-inch color LCD screen, with images displayed at the center of a black background. The image size was 13.5 cm × 11.5 cm (width × height), providing a viewing angle of approximately 9.6 × 8.2 degrees. The experimental protocol was implemented using E-prime 3.0, and the experiment comprised three distinct parts.

The induction of emotions entailed the abrupt and entirely arbitrary display of terrifying images on a screen, replicating the sudden visual transitions typical of horror films or video games. Participants were not provided with prior notice of the presence of such alarming stimuli. Their role did not entail reacting to these images; instead, they were instructed to maintain focus on the primary task: evaluating their self-focused and other-focused empathy toward the subsequent painful or non-painful face images, as detailed in the empathy measurement section. The emotional induction process involved the rapid presentation of images on the screen at random intervals during the task. Participants were assigned randomly to either the fear group or the control group. Those in the fear group were shown randomly appearing fearful images, while participants in the control group were presented with non-fearful cartoon animal images that were conceptually related (e.g., toy fish). Non-fearful cartoon animal images representing non-threatening counterparts (e.g., toy fish vs. shark) to ensure perceptual parity without fear elicitation.

Our experimental design included the following predictors: Between-Subject Factor—incidental fear (fear vs. control), with participants assigned to one condition (no counterbalancing); Within-Subject Factors—race/group membership (ingroup vs. outgroup) and image type (pain vs. non-pain), with all participants exposed to all within-subject combinations; Fear Induction Details—fearful images (sharks, spiders from IAPS) were presented in 50% of trials (16/32 per block), randomized with non-fearful cartoon animals (control group). In the fear group, fearful images (sharks, spiders from IAPS) were presented as primes in all trials. In the control group, non-fearful cartoon animal images (e.g., toy fish) were presented as primes in all trials. Each prime lasted 1000 ms, with jittered intertrial intervals (800–1200 ms) to avoid predictability. Each trial had a 50% probability of presenting a fear-evoking or non-fear-evoking picture.

In measuring empathy, participants provided responses to two theoretically distinct questions after viewing each stimulus image, adapted from established protocols (Richins et al., 2021). Self-focused empathy—reflecting personal emotional engagement with the observed pain—was operationalized via the prompt “To what extent was the event painful for you to witness?” Other-focused empathy (compassion), indexing concern for the target’s welfare, was assessed with “To what extent did you feel bad for the target?” Responses were recorded using a 7-point visual analogue scale (VAS), a widely validated method in emotion research, where 1 indicated “not at all” and 7 indicated “very much so.” Participants registered their ratings via a standard keyboard, with response windows capped at 4000 ms to ensure consistency across trials. This dual-measure approach explicitly differentiated self-oriented vs. other-oriented empathic responses, aligning with contemporary frameworks in intergroup empathy research.

At the outset, an 800 ms fixation point was displayed, followed by the random appearance of either a fearful or non-fearful image lasting 1000 ms. Subsequently, painful or non-painful stimuli images were presented for 1500 ms. Participants were then required to rate their personal pain experience on a set of 7-point scales, ranging from 1 (not at all) to 7 (very much so), and to assess the perceived pain of the faces on the screen using the same scale. Two blocks existed in the experiment, providing subjects with the chance to rest (Figure 1).

#### 2.1.5. Data Analysis

Outliers were defined as values ± 3 SDs from the condition mean. No trials or participants were excluded due to outliers. Normality checks (Shapiro–Wilk tests) confirmed that empathy scores met assumptions for parametric tests (*p* > 0.05 for all conditions).

Two (Incidental Fear: between-subjects) × Two (Race: within-subjects) × Two (Image Type: within-subjects) mixed ANOVA for empathy and IOS scores, complemented by independent-samples *t*-tests for trait empathy comparisons, were carried out. Paired *t*-tests compared pre- and post-test fear scores.

### 2.2. Results

#### 2.2.1. Manipulation Check

A 2 (Group: fear vs. control) × 2 (Time: pre-test vs. post-test) mixed ANOVA was conducted to examine the interaction between group and measurement moment on fear ratings. The analysis revealed a significant interaction effect, where *F*(1, 51) = 5.23, *p* = 0.026, *ƞ_p_*^2^ = 0.09, indicating that the pre-to-post change in fear differed between groups. Follow-up paired *t*-tests confirmed the nature of this interaction:

Paired *t*-tests compared pre- and post-test fear scores. Normality was confirmed via Shapiro–Wilk tests (*p* > 0.10). There was a significant difference between the pre- and post-tests in the fear condition (experimental group), where *t* (26) = −2.50, *p* = 0.019, *d* = −0.481, 95%CI = [−0.876, −0.078]. Participants in the post-test (3.11 ± 1.67) reported feeling more afraid than in the pre-test (2.22 ± 1.805). There was no difference between the pre- and post-tests in the control group, where *t* (25) = −1.80, *p* = 0.083, *d* = −0.35, 95%CI = [−0.747, 0.046]. There was no significant difference between the fear group and the control group in the pre-test, where *t* (24) = 0.78, *p* = 0.440, *d* = 0.15, 95%CI = [−0.239, 0.550]. There was a significant difference between the fear group and the control group in the post-test, where *t* (24) = 2.52, *p* = 0.019, *d* = 0.50, 95%CI = [0.083, 0.917]. Participants in the fear group (3.20 ± 1.70) reported feeling more afraid than those in the control group (2.28 ± 1.51). No changes occurred in non-target emotions (sadness, anger, disgust; all *ps* > 0.05), confirming fear-specific induction.

All *t*-tests were two-sided, consistent with standard practices in psychological research.

#### 2.2.2. Self-Target Overlap

To investigate fear’s effect on self-target overlap (IOS scores), we ran a 2 (race type: same race vs. other race) × 2 (incidental fear: fear vs. control) repeated-measures analysis of variance (ANOVA).

There was no main effect of race type, where *F*(1, 51) = 3.76, *p =* 0.060, *ƞ_p_*^2^ = 0.06 (*M_Same race_* ± *SD* = 5.05 ± 0.25; *M_Other race_* ± *SD* = 4.41 ± 0.27), with no main effect of fear, *F*(1, 51) = 3.29, *p =* 0.058, *ƞ_p_*^2^ = 0.07 (*M_fear_* ± *SD* = 4.85 ± 0.28; *M_control_* ± *SD* = 4.61 ± 0.27). There was an interaction between fear and race, *F*(1, 51) = 4.71, *p* = 0.035, *ƞ_p_*^2^ = 0.08. Whereas those exposed to fearful images reported less overlap with the other races (3.92 ± 0.37) compared to Same race faces (5.29 ± 0.35), *p* = 0.005, those exposed to non-fearful images reported equal levels of other-focused empathy for both groups, *p = 0*.872.

#### 2.2.3. IRI-C

We used an independent sample t-test to compare whether there was a difference in trait empathy between the two groups. The results showed that there was no significant difference in scores between the two groups, *t* = −0.77, *p* = 0.44, *d* = −0.41, 95%CI [−7.31, 3.22].

#### 2.2.4. Empathy Task

To investigate the effects of fear on self-focused and other-focused empathy, we ran a 2 (incidental fear: fear vs. control) × 2 (race: same race vs. other race) × 2 (image type: pain vs. non-pain) repeated-measures ANOVA.

**Figure 2:**
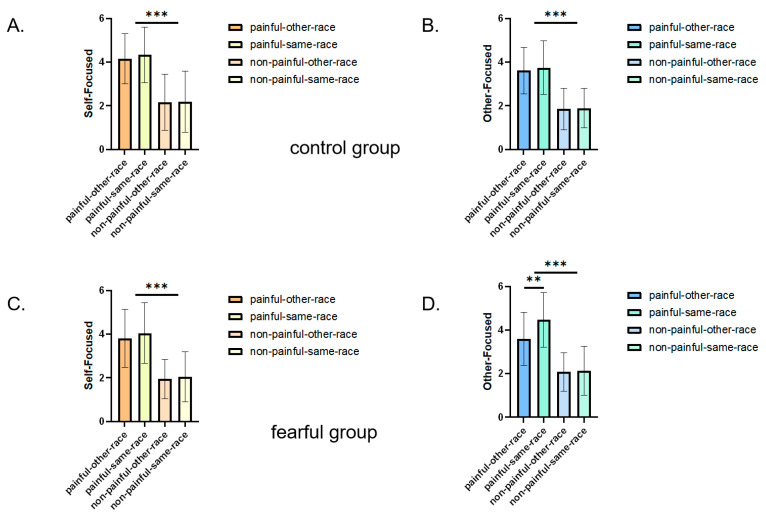
Results from experiment 1,depicting self-reported intergroup empathy in the fearful versus control group: (**A**) control group (self-focused), (**B**) control group (other-focused), (**C**) fearful group (self-focused), (**D**) fearful group (other-focused). Error bars represent standard errors of the means; *** *p* < 0.001, ** *p* < 0.01.

The results are shown in Figure 2A,B and Table 1.

#### 2.2.5. Self-Focused Empathy

The results showed a main effect of race emerged for self-focused empathy scores, *F*(1, 51) = 101.96, *p* < 0.001, *ƞ_p_*^2^ = 0.21, 95%CI = [1.606, 2.402]. Participants provided higher self-focused empathy ratings for painful faces (*M* = 4.08 ± 0.17) than non-painful faces (*M* = 2.08 ± 0.16). A main effect of race emerged, *F*(1, 51) = 6.48, *p* = 0.014, *ƞ_p_*^2^ = 0.08, with higher ratings for same race faces (*M* = 3.15 ± 0.14) vs. other race faces (*M* = 3.019 ± 0.131). An image type × race interaction, *F*(1, 51) = 4.28, *p* = 0.044, *ƞ_p_*^2^ = 0.01, revealed that for painful images, same race faces (*M* = 4.19 ± 0.18) elicited significantly higher self-focused empathy than other race faces (*M* = 3.98 ± 0.17), where *p* = 0.002, though both groups showed higher ratings for painful vs. non-painful stimuli (*ps* < 0.001).

There was no main effect of fear, where *F*(1, 51) = 0.79, *p* = 0.377, *ƞ_p_*^2^ = 0.02, 95%CI = [−0.786, 0.303], nor any interaction between fear and image type, where *F*(1, 51) = 0.12, *p* = 0.727, *ƞ_p_*^2^ = 0.01. There was no interaction between fear and race type, *F*(1, 51) = 0.41, *p* = 0.520, *ƞ_p_*^2^ = 0.01. The interaction of fear condition × race type × image type was no significant, *F*(1, 51) = 0.01, *p* = 0.917, *ƞ_p_*^2^ = 0.01.

#### 2.2.6. Other-Focused Empathy

A main effect of image type, *F*(1, 51) = 109.78, *p* < 0.001, *ƞ_p_*^2^ = 0.25, indicated higher other-focused empathy ratings for painful faces (*M* = 3.854 ± 0.139) vs. non-painful faces (*M* = 1.99 ± 0.12). A main effect of race, *F*(1, 51) = 9.07, *p* = 0.004, *ƞ_p_*^2^ = 0.15, showed greater empathy for same race faces (*M* = 3.05 ± 0.11) than other race faces (*M* = 2.78 ± 0.11). There was no main effect of fear, *F*(1, 51) = 2.09, *p* = 0.153, *ƞ_p_*^2^ = 0.08, 95%CI = [−0.697, 0.113].

There was an interaction between image type and race, *F*(1, 51) = 5.91, *p* = 0.02, *ƞ_p_*^2^ = 0.20. Pain scores for both same race and other race faces in the pain pictures were significantly higher than those in the non-pain pictures, with all *ps* < 0.001. Participants reported feeling more other-focused empathy, when the target in pain was same race face (4.10 ± 0.17), compared to other race face (3.60 ± 0.15), *p* = 0.005, 95%CI = [0.157, 0.848].

Furthermore there was an interaction between fear and race, *F*(1, 51) = 4.31, *p* = 0.043, *ƞ_p_*^2^ = 0.08: whereas those exposed to fearful images reported significantly less other-focused empathy for other race faces (2.83 ± 0.15) compared to Same race faces (3.30 ± 0.15), *p* = 0.001, 95%CI = [0.208, 0.721], those exposed to non-fearful images reported equal levels of other-focused empathy for both groups, *p =* 0.515, 95%CI = [−0.176, 0.347]. For Same race faces, people exposed to fearful imaged reported more other-focused empathy (3.30 ± 0.15) than exposed to non-fearful images (2.835 ± 0.156), *p* = 0.034, 95%CI = [0.039, 0.924]. For other race faces, there was no difference between fearful group and control group, *p* = 0.882, 95%CI = [−0.345, 0.549]. The interaction of fear condition × race type × image type was significant, *F*(1, 51) = 3.63, *p* = 0.06, *ƞ_p_*^2^ = 0.07. In the fear group, the pain score of the native race was higher than that of the other ethnic faces, *p* = 0.001, 95%CI *=* [0.387, 1.355]. For native faces, the pain score was higher in the fear group than in the control group, *p* = 0.038, 95%CI *=* [0.038, 1.403]. Regardless of ethnicity, it was the pain face that scored higher than the non-pain face, all *ps* < 0.001.

### 2.3. Discussion

The results indicated that incidental fear influenced other-focused empathy, with participants showing reduced empathy towards out-group members upon viewing alarming images. Intergroup biases in other-focused empathy were evident only during fear experiences, whereas self-focused empathy showed no such association. The IOS scale revealed that incidental fear increased psychological distance from out-group members, which we interpret as potentially influencing empathetic responses, given theoretical links between psychological distance and empathy. The study further examined intergroup materials featuring racially diverse faces, exploring the potential extension of accidental emotions’ influence on pain empathy from external to artificially delineated social groups. Experiment 2 used Same race facial stimuli categorized as own school or other school, hypothesizing reduced pain empathy towards the “other school” group due to incidental fear emotions.

## 3. Experiment 2

### 3.1. Method

#### 3.1.1. Participants

G*Power 3.1 was used with an ANOVA (repeated measures, within-between interaction) setting. The input parameters were as follows: *f* = 0.25, *α* = 0.05 and power = 0.80, yielding a minimum *N* = 24. To account for potential attrition and design complexity, we recruited 52 participants. (24 females, aged 20.66 ± 1.59 years). Participants were randomly assigned to the fear group (14 females, 11 males, aged 20.84 ± 1.79 years) and the control group (10 females, 15 males, aged 20.48 ± 1.66 years). Participants were right-handed individuals with normal or corrected vision, who had no history of chronic or mental illness. All participants provided written, informed consent before beginning experimentation.

#### 3.1.2. Stimulus

We selected 64 images (Same race faces) from IPAS, comprising both pain and non-pain stimuli, evenly distributed between male and female subjects. A total of 64 trials, 50% depicting painful events (32), and 25% per group membership (16), were presented in two blocks.

#### 3.1.3. Measure

Similar to Experiment 1, participants were asked to first perform a self-emotion assessment and IRI-C, then respond to two empathy questions for each picture of a person, and finally rate their closeness on an IOS scale.

#### 3.1.4. Procedure

All the faces the subjects saw in the experimental materials were of the same race, and they were divided into their own school (in-group) and other school (out-group). The experimental process for this study was identical to that of Experiment 1. Before each face stimulus, a text-based cue (e.g., “Zhejiang Normal University Student” for in-group or “Jiangxi Normal University Student” for out-group) was displayed for 1000 ms on the screen. These cues were accompanied by university logos (e.g., Zhejiang Normal University’s emblem for in-group; Jiangxi Normal University’s emblem for out-group) to reinforce group identity visually. Since all our participants were from Zhejiang Normal University, Jiangxi Normal University was considered an out-group for them. These two universities were chosen because they were geographically distinct (located in different provinces) yet comparable in academic standing and student demographics, minimizing confounding variables related to institutional status—consistent with the aim to create ecologically valid yet controlled intergroup distinctions (as noted in the general discussion of using real-world institutional identities).

This manipulation aligned with social identity theory, where minimal group distinctions (even artificial ones) could elicit in-group favoritism and out-group bias. By anchoring group membership to institutional affiliation, we replicated real-world intergroup dynamics in a controlled setting.

#### 3.1.5. Data Analysis

Two (Incidental Fear: between-subjects) × Two (Group Membership: within-subjects) × Two (Image Type: within-subjects) mixed ANOVA was carried out, following the same logic as Experiment 1.

### 3.2. Results

#### 3.2.1. Manipulation Check

A 2 (Group: fear vs. control) × 2 (Time: pre-test vs. post-test) mixed ANOVA was conducted to examine the interaction between group and measurement moment on fear ratings. The analysis revealed a significant interaction effect, where *F*(1, 48) = 12.86, *p* < 0.001, *ƞ_p_*^2^ = 0.21, indicating that the pre-to-post change in fear differed between groups. Follow-up paired *t*-tests confirmed the nature of this interaction.

Paired *t* tests compared pre- and post-test fear scores. Normality was confirmed via Shapiro–Wilk tests (*p* > 0.10). There was a significant difference between the pre- and post-tests in the fear condition (experimental group), *t* (24) = −6.41, *p* < 0.001, *d* = −1.28, 95%CI = [−1.809, −0,743]. Participants in the post-test reported feeling (3.52 ± 1.66) more afraid than in the pre-test (1.68 ± 1.28). There was no difference between the pre- and post-tests in the control group, *t* (24) = −0.96, *p* = 0.343, *d* = −0.19, 95%CI = [−0.587, 0.204]. There was no significant difference between the fear group and the control group in the pre-test, *t* (24) = −0.67, *p* = 0.507, *d* = −0.13, 95%CI = [−0.527, 0.260]. There was a significant difference between the fear group and the control group in the post-test, *t* (24) = 2.56, *p* = 0.015, *d* = 0.52, 95%CI = [0.101, 0.938]. Participants in the experimental group (3.52 ± 1.66) reported feeling more afraid than those in the control group (2.44 ± 1.22).

No changes occurred in non-target emotions (sadness, anger, disgust; all *ps* > 0.05), confirming fear-specific induction.

#### 3.2.2. IRI-C

We used an independent-sample *t*-test to compare whether there was a difference in trait empathy between the two groups. The results showed that there was no significant difference in scores between the two groups, *t* = −0.89, *p* = 0.37, *d* = −0.52, 95%CI = [−7.76, 2.95].

#### 3.2.3. Self-Target Overlap

To investigate fear’s effect on self-target overlap (IOS scores), we ran a 2 (group membership: in-group vs. out-group) × 2 (incidental fear: fear vs. control) repeated-measures analysis of variance (ANOVA).

There was no main effect of fear, *F*(1, 48) = 0.15, *p* = 0.902, *ƞ_p_*^2^ = 0.09, *M_fea_*_r_ ± *SD* = 3.02 ± 0.34; *M_control_* ± *SD* = 2.96 ± 0.34. Participants reported more overlap with the in-group (3.56 ± 0.32), compared to the out-group (2.42 ± 0.24), *F*(1, 48) = 13.60, *p* = 0.001, *ƞ_p_*^2^ = 0.22. There was a marginal interaction between fear and race, where *F*(1, 48) = 3.52, *p* = 0.067, *ƞ_p_*^2^ = 0.08, whereas those exposed to fearful images reported less overlap with the out-group (2.16 ± 0.34) compared to the in-group (3.88 ± 0.46), with *p* = 0.001. Those exposed to non-fearful images reported equal levels of other-focused empathy for both groups, where *p* = 0.206.

#### 3.2.4. Empathy Task

**Figure 3:**
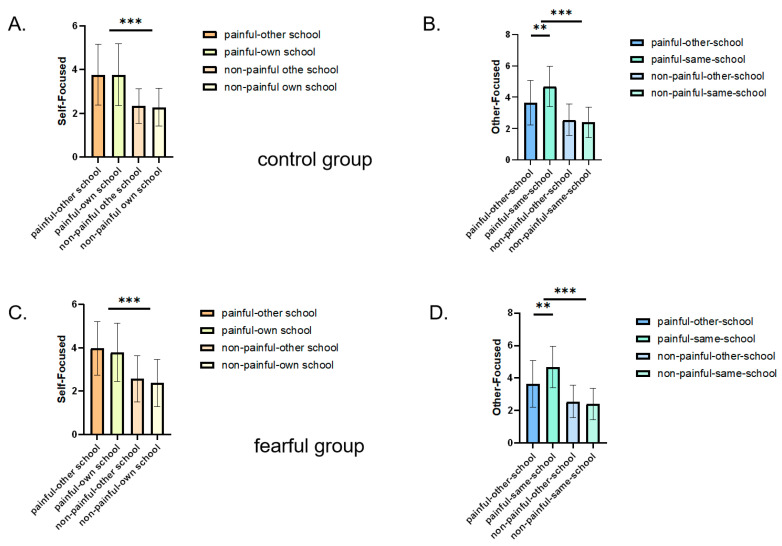
Results from experiment 2, depicting self-reported intergroup empathy in the fearful versus control group: (**A**) control group (self-focused), (**B**) control group (other-focused), (**C**) fearful group (self-focused), (**D**) fearful group (other-focused). Error bars represent standard errors of the means; *** *p* < 0.001, ** *p* < 0.01.

To investigate the effects of fear on intergroup, we ran a 2 × 2 × 2 mixed ANOVA. The results are shown in Figure 3 and Table 2.

#### 3.2.5. Self-Focused Empathy

The results showed the main effect of image type, *F*(1, 48) = 65.01, *p* < 0.001, *ƞ_p_*^2^ = 0.20, 95%CI = [−7.553, −1.078]. For pictures of painful faces, the rating of pain (3.82 ± 0.18) was significantly higher than that regarding non-painful pictures (2.388 ± 0.133). The results showed the main effect of membership, *F*(1, 48) = 5.53, *p* = 0.023, *ƞ_p_*^2^ = 0.11, 95%CI = [−0.200, −0.016]. For pictures of group membership, the rating of the in-group (3.16 ± 0.13) was significantly higher than that regarding the out-group (3.05 ± 0.14).

There was no main effect of fear, *F*(1, 48) = 0.25, *p* = 0.61, *ƞ_p_*^2^ = 0.01, 95%CI = [−0.411, 0.688], nor any interaction between fear and image type, *F*(1, 48) = 0.02, *p* = 0.901, *ƞ_p_*^2^ = 0.01. There was no interaction between fear and group membership, *F*(1, 48) = 0.36, *p* = 0.548, *ƞ_p_*^2^ = 0.01. The interaction of fear condition × group membership × image type was no significant, where *F*(1, 48) = 0.22, *p* = 0.638, *ƞ_p_*^2^ = 0.01.

#### 3.2.6. Other-Focused Empathy

The results showed a main effect of image type, *F*(1, 48) = 90.11, *p* < 0.001, *ƞ_p_*^2^ = 0.27, 95%CI = [−1.776, −1.156]. For pictures of painful faces, the rating of pain (3.85 ± 0.13) was significantly higher than that for non-painful pictures (2.38 ± 0.12). There was no main effect of group membership, where *F*(1, 48) = 3.39, *p =* 0.073, *ƞ_p_*^2^ = 0.07, 95%CI = [−0.019, 0.439], no main effect of fear, where *F*(1, 48) = 3.67, *p =* 0.062, *ƞ_p_*^2^ = 0.07, 95%CI = [−0.815, 0.020].

There was an interaction between image type and group membership, *F*(1, 48) = 6.85, *p* = 0.01, *ƞ_p_*^2^ = 0.21. Pain scores for both own school and other school in the pain pictures were significantly higher than those in the non-pain pictures, with all *ps* < 0.001. For pain pictures, the face pictures from the participant’s own school induced a greater pain score than the face pictures from other schools, where *p* = 0.02, 95%CI = [0.070, 0.957].

Furthermore there was an interaction between fear and group membership, where *F*(1, 48) = 4.21, *p* = 0.047, *ƞ_p_*^2^ = 0.08, whereas those exposed to fearful images reported significantly less other-focused empathy for other school faces (3.09 ± 0.18) compared to own school faces (3.54 ± 0.15), where *p* = 0.008, 95%CI = [−0.768, 0.120]. Those exposed to control images reported equal levels of other-focused empathy for both groups, where *p =* 0.882. For own school faces, people exposed to fearful images reported more empathy (3.54 ± 0.15) than the control (3.09 ± 0.18), *p* = 0.005, 95%CI = [0.195, 1.067]. For other school faces, there was no difference between the fearful group and control group, where *p* = 0.882.

The interaction of group membership × image type was significant, where *F* (1, 48) = 6.37, *p* = 0.015 and *ƞ_p_*^2^ = 0.18 for painful pictures; the own school reported more empathy in the fear condition (4.11 ± 0.17) than in the control condition (3.60 ± 0.18), *p* = 0.024, 95%CI = [0.070, 0.957]. For non-painful pictures, there was no difference between the in-group (2.43 ± 0.13) and the out-group (2.43 ± 0.12), *p* = 0.15, 95%CI = [−0.035, 0.223]. Whether it was the school or the outside school, the score of pain pictures was significantly higher than that of non-pain pictures, with all *ps* < 0.001.

The interaction of image type × group membership × image type × fear was significant, *F* (1, 48) = 6.36, *p* = 0.015, *ƞ_p_*^2^ = 0.23. For the faces of this school, the pain score in the fear group was significantly higher than that in the control group, *p* < 0.001, 95%CI = [0.481, 1.804]. For painful images, in-group faces elicited higher pain scores than out-group faces in the fear condition (*p* = 0.02), 95%CI = [0.412, 1.668]. None of the other effects were significant.

### 3.3. Discussion

The present findings indicate that the difference in empathy for ingroup and outgroup members’ pain primarily stems from other-focused empathy, with accidental fear significantly diminishing group empathy levels. The findings of Experiment 1 are applicable to socially segregated groups, demonstrating the consistent impact of incidental fear in diminishing empathy for out-group pain.

## 4. General Discussion

This study examines how incidental fear shapes empathy for in-group and out-group pain across two experiments, advancing understanding of intergroup empathy bias. Unlike prior research focusing on integrated emotions (e.g., chronic fear tied to group contexts), we isolate the effect of incidental fear—a transient, context-irrelevant emotional state rarely studied in intergroup dynamics.

Our key findings are as follows: First, even brief, unrelated fear (e.g., induced by aversive animal images) selectively reduces other-focused empathy for out-group pain, distinguishing it from deliberative emotional processes. Experiment 1 demonstrates reduced empathy for other-race (out-group) pain among Chinese participants under fear. Building upon this, Experiment 2 shows that even simple artificial divisions (e.g., “own school” vs. “other school”) elicit similar variations in pain empathy, revealing that incidental fear amplifies empathy bias even in minimally defined groups. This bridges natural (racial) and artificial (school-based) group divisions, underscoring that fear’s capacity to rigidify intergroup boundaries transcends categorization type—extending social identity theory to transient emotions and artificial groups.

Notably, our findings also reveal a dissociation: incidental fear selectively impacts other-focused empathy while leaving self-focused empathy unaltered. Empathy involves both affective sharing (self-focused) and cognitive perspective-taking (other-focused), supported by dissociable neural systems (Mocaiber et al., 2011). Neurobiologically, self-focused empathy—indexing personal discomfort in witnessing pain—relies on sensorimotor pathways (e.g., anterior insula, mirror neuron systems) that process visceral and sensory features of pain stimuli, the activation of which is anchored in the stimulus’s inherent salience (e.g., high pain intensity ratings for both groups in Experiment 1). These low-level, automatic processes are less susceptible to fear modulation, as they reflect direct sensory-affective simulation rather than social-cognitive evaluation of group membership. In contrast, other-focused empathy engages mentalizing networks (e.g., medial prefrontal cortex, temporoparietal junction), which assess others’ subjective states and intergroup relevance—a process amplified by fear-induced threat vigilance and psychological distancing (Aron et al., 1992; Navarrete et al., 2012). Methodologically, the null effect on self-focused empathy may arise from measurement design: self-focused ratings (“To what extent was the event painful for you to witness?”) conflate visceral reactivity with vicarious affect, prioritizing the stimulus’s physical properties (e.g., needle vs. cotton swab) over contextual emotions like fear. Additionally, the fear-inducing stimuli (e.g., spiders, sharks) used in this study lack direct relevance to the pain context, creating a weaker emotional link to self-focused empathy, which is more tightly coupled to first-hand pain experience. By contrast, other-focused empathy—a social cognitive judgment—is more vulnerable to fear-induced biases in intergroup perception, as demonstrated by the enhanced empathy bias in the fear condition (Richins et al., 2021).

Our findings are consistent with previous research (Richins et al., 2021), but also provide some important new contributions to the existing literature. To begin with, Richins et al.’s (2021) study selected straight pictures of aversion and fear and has not yet rated them as material, but the present study enhanced the precision and replicability of the research findings by objectively evaluating their validity and arousal. Then, although Richins’ study primarily examined the manipulation of in-group and out-group dynamics through social classification, there is a lack of research on how incidental fear influences pain empathy among various ethnic groups. Nevertheless, our research encompasses a variety of ethnicities. By incorporating diverse ethnicities in the study, we not only confirm the consistency of the findings but also expand the scope of the observed effects. Richins et al. (2021) reported *η_p_*^2^ = 0.12 for fear’s impact on out-group empathy, whereas our Experiment 1 found *η_p_*^2^ = 0.08 for the fear × race interaction. This suggests our effects are modest but consistent with previous social categorization studies. Differences in magnitude may stem from methodological variations (e.g., ethnic vs. social group distinctions).

Beyond these core results, our study makes broader contributions, particularly regarding generalizability across cultural contexts. Nearly all prior studies on fear and intergroup bias have been conducted with Western samples (e.g., European or American participants; e.g., (Navarrete), leaving the generalizability of findings to non-Western contexts untested. Our use of Chinese participants addresses this gap, demonstrating that incidental fear intensifies empathy bias in a collectivist cultural context—where group identities may be more salient and intergroup boundaries differently constructed compared to individualist societies. Experiment 1 shows reduced empathy for other race (out-group) pain among Chinese participants under fear, extending Western findings to a non-Western population and challenging assumptions of cultural universality.

We further explore how incidental fear affects intergroup attitudes, finding that fear stimuli (e.g., spiders, snakes) enhance out-group distancing via reduced self-other overlap. The self-others overlap scale (Aron et al., 1992), which measures perceived similarity with others, revealed that fear exacerbates psychological disparities between groups, diminishing out-group empathy—consistent with prior work linking self-other overlap to empathic accuracy. Specifically, fear activates threat-related neural and cognitive processes (e.g., heightened vigilance, amygdala engagement) that amplify psychological distance toward out-groups. Additionally, our findings indicate that fear increases psychological distance from out-groups, thereby intensifying prejudice toward them. This distancing reduces self-other overlap, a key substrate for other-focused empathy involving mentalizing networks (Frith & Frith, 2003) (e.g., mPFC, temporoparietal junction). By disrupting shared representation of others’ states, fear-driven psychological distance diminishes empathic concern for out-group pain—aligning with social identity theory (Singer et al., 2004), where fear exacerbates intergroup boundaries by priming defensive in-group favoritism.

Additionally, our findings highlight the role of situational factors like emotions in shaping empathy bias (Bodenhausen et al., 1994, 2001; Phaf & Rotteveel, 2005). Incidental fear enhances group bias, diminishing pain empathy toward other races and extending to socially classified contexts. Fear’s role in survival—signaling perceived threats to resources (Nudelman et al., 2022)—may render out-group fear adaptively beneficial, though it also acts as a heuristic source of bias (Kruglanski, 2010; Vohs et al., 2007), consistent with its carryover effects on judgments (Liu et al., 2016). Incidental emotions like fear prompt heuristic judgments (Kruglanski, 2010), which can bias intergroup evaluations. However, other negative states (e.g., sadness) may trigger systematic processing (Lerner et al., 2013), highlighting the need for emotion-specific frameworks. This distinction underscores why fear selectively disrupts other-focused empathy—a cognitively mediated process vulnerable to heuristic biases—while sparing sensorimotor-based self-focused empathy.

### 4.1. Limitations and Future Research

While our findings provide novel insights into incidental fear and intergroup empathy, several key limitations merit careful consideration. First, our study inferred threat perception indirectly through the Self-Other Overlap (IOS) scale rather than directly measuring perceived threat—a central construct in intergroup dynamics (Aron et al., 1992). Although fear is theoretically linked to heightened threat vigilance, which could reduce out-group empathy, the absence of direct threat ratings weakens causal inference. To address this, we recommend future research employ validated tools like the Intergroup Threat Scale to explicitly test whether threat mediates the relationship between fear, psychological distance, and empathy bias.

Second, while our use of static images enabled controlled emotional induction, this approach has limitations in ecological validity. To bridge this gap, future studies could incorporate dynamic or multimodal stimuli (e.g., videos, virtual reality) to better simulate real-world fear-inducing contexts. However, static images remain a valuable method for isolating specific emotional effects and minimizing extraneous variables, a trade-off that balances internal validity with generalizability.

Third, although we highlighted the cultural specificity of our Chinese sample, we did not directly measure cultural orientations (e.g., individualism–collectivism) or relational values. Adding measures such as the Self-Construal Scale would help quantify how cultural traits moderate the impact of fear on empathy, an important direction for cross-cultural research.

A notable scope of the current study is its focus on fear, one specific negative emotion. This choice allows for a precise examination of fear’s unique role, while acknowledging that other negative emotions (e.g., anger, disgust) may modulate intergroup empathy through distinct pathways—a fruitful direction for future research. Fear primes avoidance, anger fuels confrontational defense, and disgust prompts moral dehumanization (Hodson et al., 2013), with anger potentially enhancing in-group empathy alongside out-group hostility, while disgust may universally suppress empathy through contamination framing. Future work should deploy emotion-specific inductions (e.g., unfair scenarios for anger, pathogen cues for disgust) coupled with multimodal measures (behavioral, physiological) to dissect these pathways. Cross-cultural designs could test whether collectivist contexts amplify fear-driven in-group shielding, whereas individualist societies exacerbate anger-rooted justice biases. Such efforts would elucidate whether fear’s empathy reduction is singular or embedded within a broader affective architecture guiding intergroup dynamics, refining theories of emotion and social identity (Kruglanski, 2010).

Finally, our choice of other race faces as the out-group in Experiment 1 raises questions about generalizability. The observed empathy reduction might reflect context-specific intergroup dynamics rather than a universal phenomenon, as biases could vary based on perceived historical, socioeconomic, or cultural threat. Future work should extend this research to other racial or social out-groups—such as ethnic minorities in multicultural settings—to clarify the boundaries of incidental fear’s effects on empathy bias.

### 4.2. Theoretical and Practical Implications

Our findings advance theoretical frameworks in two critical ways. First, they extend affective heuristics theory (Kruglanski, 2010) by demonstrating that incidental fear—a transient, context-irrelevant emotion—biases empathy through automatic threat vigilance rather than deliberative appraisal. This aligns with dual-process models of empathy, where heuristic emotional states selectively impair mentalizing-dependent processes (other-focused empathy) while sparing sensorimotor simulation (self-focused empathy). Second, this study connected the social identity theory with emotion research, showing that fear amplifies intergroup boundaries not only in natural categories (race) but also in artificial groups. This supports the notion that incidental emotions interact with minimal group paradigms to rigidify ‘us vs. them’ distinctions, even in the absence of historical conflict. These insights refine models of intergroup bias by integrating transient affective states as catalysts for empathy reduction.

Practically, our results have implications for mitigating intergroup conflict and designing inclusive policies. First, media and public communication strategies should avoid incidental fear-inducing stimuli (e.g., sensationalized imagery unrelated to intergroup contexts), as these may unconsciously exacerbate empathy bias. Second, interventions aimed at fostering intergroup empathy—such as contact hypothesis programs—could benefit from emotion-regulation components to neutralize fear-driven threat perceptions. For example, mindfulness training might attenuate fear’s heuristic impact on out-group evaluations. Third, policymakers could leverage these findings to design environments that minimize incidental fear (e.g., reducing aversive cues in diverse workplaces or schools), thereby promoting equitable empathy across group lines. Such applications align with growing efforts to address systemic bias through psychologically informed frameworks.

## 5. Conclusions

This study aimed to investigate how incidental fear influences empathy for in-group versus out-group pain in Chinese participants and to test whether such effects generalize across racial and artificial social categorizations. Experiment 1 demonstrated that incidental fear reduced other-focused empathy for other race (out-group) pain but not same race (in-group) pain. Experiment 2 replicated this pattern using school-based categorization, showing diminished empathy for “other school” members under fear. Fear selectively impaired other-focused empathy (linked to mentalizing processes) but spared self-focused empathy (rooted in shared affect), consistent with dual-process models of empathy.

## Figures and Tables

**Table 1:** Experiment 1 pain empathy score (M ± SD).

	Other-Focused Empathy				Self-Focused Empathy			
Experiment 1	Pain		Non-Pain		Pain		Non-Pain	
	Other Race	Same Race	Other Race	Same Race	Other Race	Same Race	Other Race	Same Race
Fear	3.60 ± 1.21	4.47 ± 1.25	2.08 ± 0.89	2.14 ± 1.13	3.81 ± 1.34	4.05 ± 1.39	1.95 ± 0.91	2.05 ± 1.14
Control	3.61 ± 1.07	3.75 ± 1.23	1.86 ± 0.95	1.89 ± 0.90	4.16 ± 1.14	4.33 ± 1.27	2.16 ± 1.28	2.18 ± 1.41

*Note.* These tables present the means (±standard deviations) of self-focused and other-focused empathy ratings across different conditions (fear/control, other race/same race, painful/non-painful stimuli).

**Table 2:** Experiment 2 pain empathy score (*M ± SD*).

	Other-Focused Empathy				Self-Focused Empathy			
Experiment 2	Pain		Non-Pain		Pain		Non-Pain	
	Other School	Own School	Other School	Own School	Other School	Own School	Other School	Own School
Fear	3.55 ± 1.14	3.54 ± 1.03	2.32 ± 0.77	2.28 ± 0.86	3.77 ± 1.39	3.77 ± 1.41	2.34 ± 0.78	2.28 ± 0.87
Control	3.64 ± 1.43	4.68 ± 1.28	2.55 ± 1.00	2.40 ± 0.98	3.98 ± 1.23	3.79 ± 1.34	2.57 ± 1.07	2.37 ± 1.10

*Note.* These tables present the means (±standard deviations) of self-focused and other-focused empathy ratings across different conditions (fear/control, other school/own school, painful/non-painful stimuli).

## Data Availability

Data shared in manuscript. The authors confirm that all data generated or analysed during this study are included in this published article. We report how we determined our sample size, all data exclusions (if any), all manipulations, and all measures in the study.
